# Massachusetts Veterinary Association

**Published:** 1890-07

**Authors:** Austin Peters

**Affiliations:** Secretary


*Massachusetts Veterinary Association.*—This association held its regular meeting May 28th, 1890, at 19 Boylston Place, Boston. Presiding officer, President Thomas Blackwood. Members present, Drs. Blackwood, Bryden, Howard, Lee, Marshall, Peterson, J. S. Saunders, Skally, Winchester, and Charles Winslow. Honorary member, J. H. Stickney. Visitors, Dr. H. C. Ernst, M.D., Dr J. O. Whitney, M.D., Pawtucket, R. I.; Drs. Alex. Burr, M.D.V., and Charles E. Hadcock, M.D.V.

Minutes of the last meeting read and accepted. Dr. Winchester moved that the matter of Dr. K. Winslow's resignation be acted upon at a later meeting. Seconded. Carried. Papers, discussions, etc.

The meeting having been called for a discussion of rabies, to be opened by Dr. Winchester, he commenced the discussion by saying that he claimed no originality for what he had to say, but simply gave a short résumé of the views of others, quoting Ziegler, Bruckmuller, Fleming and other pathologists. He then spoke of how difficult it is for the veterinarian to make a correct diagnosis of rabies in his patients, unless he has a good history of the case, or verifies his diagnosis by experiment. He concluded by giving Dr. F S. Billings' views of the disease, especially its having but one termination and that a fatal one. The chair then called upon Dr. H. C. Ernst, of the Bacteriological Laboratory at the Harvard Medical School, to follow Dr. Winchester in the discussion.

Dr. Ernst commenced by saying that he regretted that he could not hear more from other members present on their individual views before speaking himself, and also hoped that any shortcomings on his part would be excused, as he had hardly rested from a trip to the West. He did not see how any one could doubt the existence of rabies. Most writers on pathological anatomy are ready to acknowledge that many things which they wrote a few years ago are wrong to-day. There is no doubt about the disease being in existence, and it is believed to be due to a *contagium vivum*. He said that no wonder the symptoms vary, depending, as in a disorder of the nervous system, upon the temperament of the dog, and the surrounding influences of excitement or quiet. He quoted the case of a collie dog which he owned which had recently died of rabies, a great change being noted in his disposition in a few hours, between the morning and the afternoon on the day he was taken with the malady. The dog after two or three days of suffering was chloroformed. In this case there was a history of the dog having been bitten, about three weeks previous to the time he was seized, by a strange dog passing through the street.

Dr. Ernst believes the term "hydrophobia" to be a misnomer, as the disease is not accompanied by a fear of water. We cannot judge of the disease in man by this symptom (fear of water), as the name "hydrophobia" may have an effect upon the mind of a person affecting the imagination, whereas it would not among the lower animals. He then spoke of a recent case of rabies in a man at the Massachusetts General Hospital. This man said that he was conscious at all times during the progress of the disease, but that he had not the power to resist the paroxysms which seized him, and the next moment it was all that four attendants could do to hold him down on the floor. May it not be the same in the case of the dog? Dr. Ernst believes it is, and that most of the cases of furious *rabies canina* we see result from exciting the animals, and that in most instances if the dog were left to himself it would take the form of what we call "dumb rabies." He then gave a summary of the doings of the Institute Pasteur, over 7000 persons having received the protective inoculation in four years, with a loss of only 0.67 per cent., the percentage of mortality being less from year to year as the methods of inoculation become more and more exact. There is also a greater fatality among those bitten on the head or face. As to the cauterization of bites from rabid animals the cases afterwards being treated by Pasteur, cauterization being performed with the hot iron and also various chemical caustics, of those treated in this way and afterward receiving Pasteur's protective inoculation the per cent. of deaths,was greater than in cases where the bite was left alone. Dr. Ernst thinks cauterization a mistake, but believes in strong ligation (if possible) and then sucking the wound. There is no danger of absorbing the poison by the mouth if the buccal mucous membrane is healthy.

Pasteur, in his "Annals of the Institute," prints a map showing how much more common the disease is in some localities than others in France, and also gives tables showing that hot weather has nothing to do with increasing the frequency of the disease, it generally being more common in the Winter months.

Dr. Ernst then spoke of the first case of rabies in this locality being brought to his attention by the secretary of this Association a year ago, and its increasing frequency up to the present time. He has also known of five cases of human rabies near Boston within the last four months, and more may have occurred which were not correctly diagnosed. He also said that all scientific bodies should protest against such evidence being produced as was given at a legislative hearing on muzzling dogs, at the State House last Winter.

In answer to questions asked him by different members, Dr. Ernst said that he believed in the efficacy of Pasteur's protective inoculation. That there is no way of making a positive diagnosis of rabies except by inoculation experiments. That he doubts if there be any spontaneous recovery from rabies, and if a case with symptoms of rabies recovered, he would not believe it was rabies unless it was proved in some way by inoculation experiments. He believed that if every dog in the community were muzzled for three months the disease would be stamped out; but it would be almost impossible to enforce such a law, even if it were passed.

The president then called upon Dr. J. O. Whitney, who cited a number of cases of rabies among people which he had seen, or that had been called to his attention in the neighborhood of Pawtucket; the instances given occurring at intervals from forty years ago down to the present Spring—all the cases being directly traceable to bites from rabid dogs. Dr. Whitney also spoke of the greater danger from the bite of a rabid wolf. He also compared the mortality among people protected by Pasteur's method—less than one per cent.—and the mortality among those who received no protective inoculation, where the death rate was fifteen per cent. of those bitten.

Dr. Bryden spoke of a case of rabies in a horse belonging to a client of his at Weymouth, which he was called to see several years ago.

Dr. Ernst then explained the principles of protective inoculation as employed by Pasteur, and described the difference between the old method at first used and the intensive method, which is the one resorted to now. He also said that Pasteur had probably not lost any cases that he began on in time, and that he had never had a case of blood poisoning resulting from inoculations against rabies.

Dr. Ernst and Dr. Saunders then discussed the question of the spontaneity of rabies, in which Dr. Ernst did not believe.

Dr. Winchester moved that a vote of thanks be given Dr. Ernst for attending and taking part in the discussion. Seconded and carried. The following cases were then reported. By Dr. Winchester: A case of cyanosis in a foal, the foal dying after violent exertion when a few days old, and the foramen ovale between the auricles being found perforate upon post-mortem examination. Specimen shown.

Dr. Stickney: A case in a yearling colt castrated this Spring. A few days after the operation the end of the cord was noticed hanging through the incision in the scrotum, two or three inches being visible; it gradually lengthened until it reached his hocks, and when colt was thrown to cut the cord off a second time, it became easily detached by manipulation, and a piece fifteen or eighteen inches came away. The colt afterward made a good recovery. Specimen shown in alcohol.[1]

Dr. Peters: A case of fracture of the skull in a horse. The horse—eight or nine years old sorrel gelding—was turned into a lane leading to a pasture; he ran down the lane, and finding the bars up, turned and ran back toward the barn; he fell in some way, striking his forehead with great force against a tree, causing an incomplete fracture of the skull near the junction of the frontal and parietal bones, horse dying from concussion of the brain, death being almost instantaneous. On post-mortem examination the thoracic and abdominal viscera were healthy. Specimen shown in a fresh state, autopsy having been made but a few hours previous. Meeting then adjourned.

AUSTIN PETERS, *Secretary.*

[1] The editor has recently had a similar case after castration in a cat, the neoplasm proved to be embryonic connective tissue.

